# Author Correction: Satellites reveal hotspots of global river extent change

**DOI:** 10.1038/s41467-023-38302-1

**Published:** 2023-05-05

**Authors:** Qianhan Wu, Linghong Ke, Jida Wang, Tamlin M. Pavelsky, George H. Allen, Yongwei Sheng, Xuejun Duan, Yunqiang Zhu, Jin Wu, Lei Wang, Kai Liu, Tan Chen, Wensong Zhang, Chenyu Fan, Bin Yong, Chunqiao Song

**Affiliations:** 1 Key Laboratory of Watershed Geographic Sciences, Nanjing Institute of Geography and Limnology, Chinese Academy of Sciences, Nanjing, 210008 China; 2 School of Biological Sciences and Institute for Climate and Carbon Neurality, The University of Hong Kong, Pokfulam Road, Hong Kong, China; 3 College of Hydrology and Water Resources, Hohai University, Nanjing, 210098 China; 4 State Key Laboratory of Hydrology-Water Resources and Hydraulic Engineering, Hohai University, Nanjing, 210098 China; 5 Department of Geography and Geospatial Sciences, Kansas State University, Manhattan, KS 66506 USA; 6 Department of Earth, Marine and Environmental Sciences, University of North Carolina, Chapel Hill, NC USA; 7 Department of Geosciences, Virginia Polytechnic Institute and State University, Blacksburg, VA USA; 8 Department of Geography, University of California, Los Angeles, CA 90095 USA; 9 State Key Laboratory of Resources and Environmental Information System, Institute of Geographic Sciences and Natural Resources Research, Chinese Academy of Sciences, Beijing, 100101 China; 10 School of Geography and Ocean Science, Nanjing University, Nanjing, 210023 China; 11 University of Chinese Academy of Sciences, Nanjing (UCASNJ), Nanjing, 211135 China

**Keywords:** Hydrology, Environmental impact, Hydrology

Correction to: *Nature Communications* 10.1038/s41467-023-37061-3, published online 22 March 2023

The original version of this Article contained an error in Fig. 5, in which the River names Chulym River (5c), Sutlej River (5h) and Paraná River (5f) were followed by basin names. This was un-necessary and the basin names have been removed. This has been corrected in both the PDF and HTML versions of the Article.

