# Supplementary material for: Satellites reveal hotspots of global river extent change
Source: Nat Commun. 2023 Mar 22;14:1587. doi: 10.1038/s41467-023-37061-3 (PMC10033638; doi:10.1038/s41467-023-37061-3)
Supplement: Supplementary file 1 — SUPPLEMENTARY INFORMATION [file 41467_2023_37061_MOESM1_ESM.pdf]

Manuscript (Article) to *Nature Communications*

Satellites reveal hotspots of global river extent change

Qianhan Wu[1, 2, 12], Linghong Ke[3, 4, 12], Jida Wang[5], Tamlin M. Pavelsky[6], George H. Allen[7], Yongwei Sheng[8], Xuejun Duan[1], Yunqiang Zhu[9], Jin Wu[2], Lei Wang[1], Kai Liu[1], Tan Chen[1], Wensong Zhang[10], Chenyu Fan[1], Bin Yong[3, 4], Chunqiao Song[1, 11]*

[1] Key Laboratory of Watershed Geographic Sciences, Nanjing Institute of Geography and Limnology, Chinese Academy of Sciences, Nanjing 210008, China.

[2] School of Biological Sciences and Institute for Climate and Carbon Neurality, The University of Hong Kong, Pokfulam Road, Hong Kong, China.

[3] College of Hydrology and Water Resources, Hohai University, Nanjing 210098, China.

[4] State Key Laboratory of Hydrology-Water Resources and Hydraulic Engineering, Hohai University, Nanjing 210098, China

[5] Department of Geography and Geospatial Sciences, Kansas State University, Manhattan, KS 66506, USA.

[6] Department of Earth, Marine and Environmental Sciences, University of North Carolina, Chapel Hill, NC, USA.

[7] Department of Geosciences, Virginia Polytechnic Institute and State University, Blacksburg, VA, USA.

[8] Department of Geography, University of California, Los Angeles, CA 90095, USA.

[9] State Key Laboratory of Resources and Environmental Information System, Institute of Geographic Sciences and Natural Resources Research, Chinese Academy of Sciences, Beijing 100101, China.

[10] School of Geography and Ocean Science, Nanjing University, Nanjing 210023, China.

[11] University of Chinese Academy of Sciences, Nanjing (UCASNJ), Nanjing 211135, China

[12] These authors contributed equally: Qianhan Wu, Linghong Ke.

* *Correspondence to* Chunqiao Song (Email: cqsong@niglas.ac.cn)

Supplementary text

Comparison of OCI between the Pekel's GSW and the Pickens's GLAD datasets

We compared the pattern of the OCI map derived from the GSW data with that from the Global Land Analysis and Discovery (GLAD) [1]. For the comparison, we selected fifteen cases covering different types of river extent changes (e.g., stable, widening, narrowing) and morphological dynamics of braided or meandering rivers within different climate zones. Given that GLAD covers the period of 1999-2018 rather than 1984-2018, for the sake of comparison we split the period of GLAD into two 10-year epochs, i.e., 1999–2008 and 2009–2018, and regenerated the GSW OCI between the two 10-year epochs based on the monthly history layer from the GSW dataset[2]. First, we created the surface water occurrence map for each homologous pair of months in both epochs, by dividing the number of water detection by valid observation. Then the occurrence difference between the two epochs was imputed and averaged as the change intensity result (Supplementary Fig. S19). We obtained the GLAD OCI data using the same method by averaging the available percent of water monthly observation[1] for two epochs. The pattern of water inundation frequency changes derived from the GLAD OCI map is similar to that from the GSW OCI (Supplementary Fig. S19), although the two datasets are based on different algorithms and assumptions. In Supplementary Fig. S19, the differences between GSW OCI and GLAD OCI are mostly within 10%, except for a higher difference of 30-100% in a few river segments where the water frequency has changed considerably.

Supplementary figures and tables

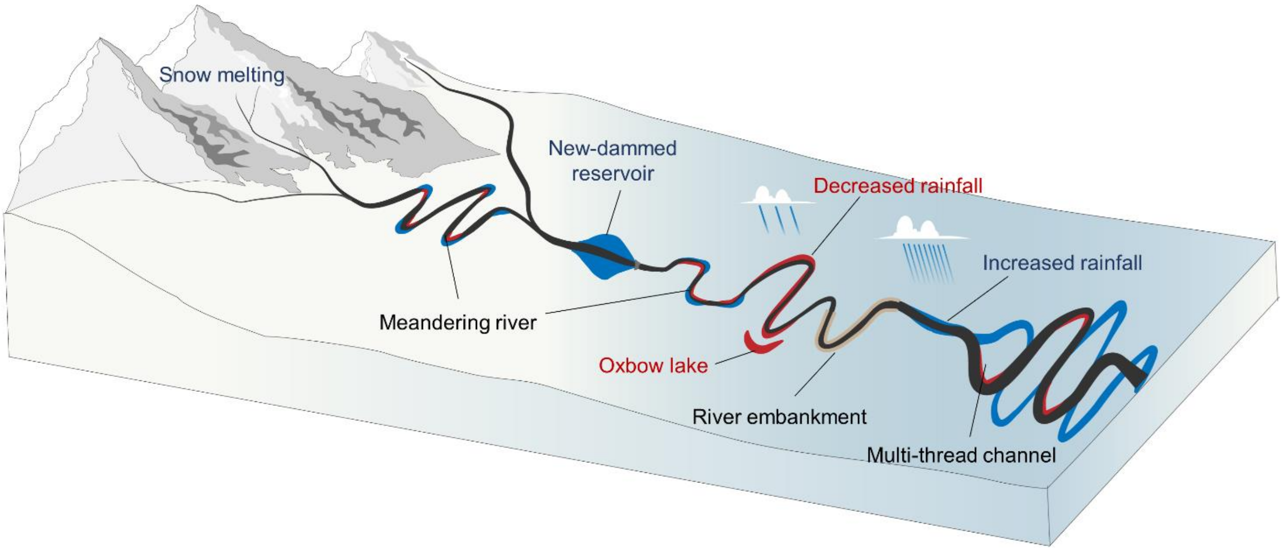

Fig. S1 | Schematic chart of typical scenarios of changes in river flow extents. Blue/red flows denote areas with increased/decreased intensity of water inundation.

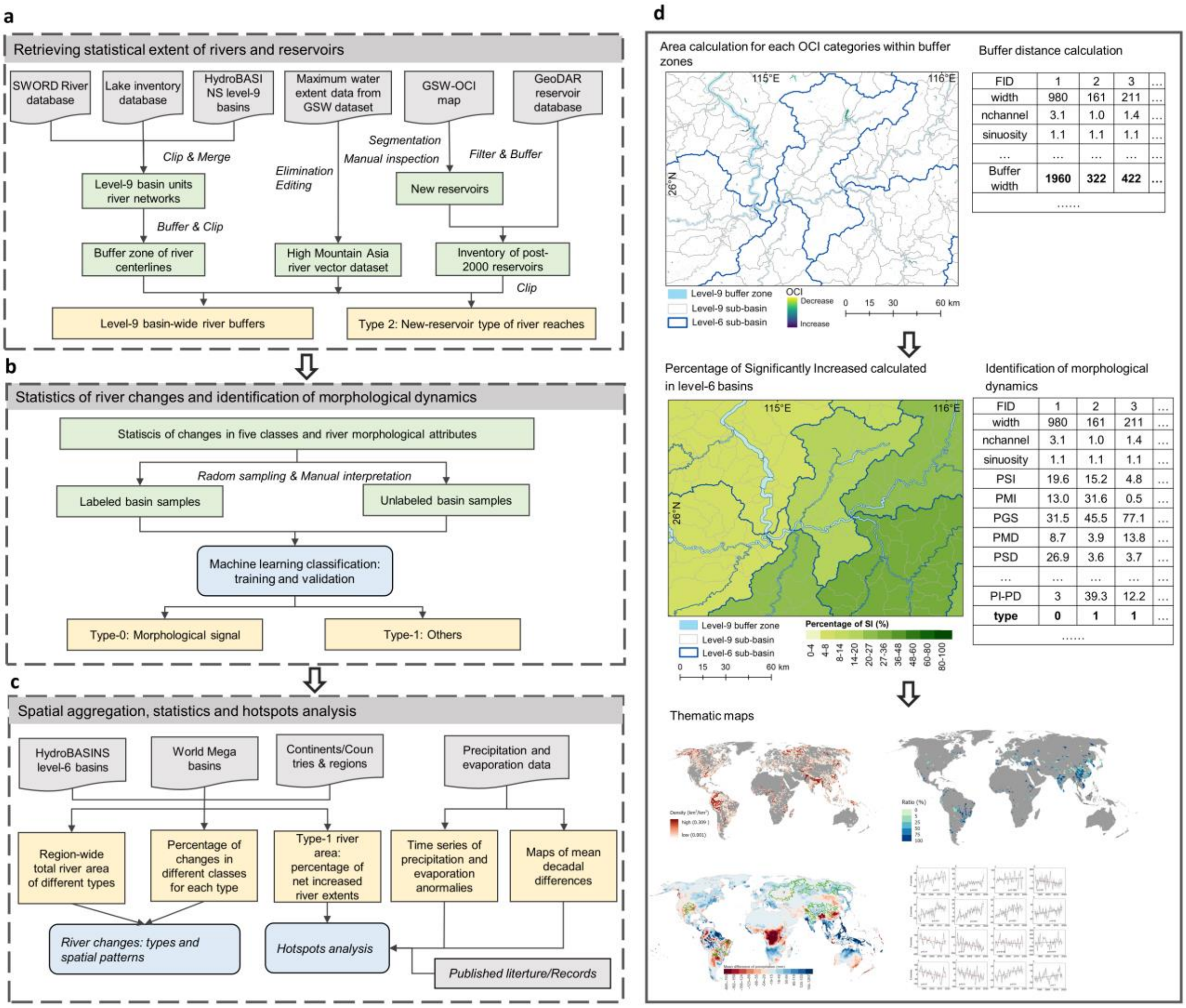

FID	1	2	3	...
width	980	161	211	...
nchannel	3.1	1.0	1.4	...
sinuosity	1.1	1.1	1.1	...
...	...	...	...	...
Buffer width	**1960**	**322**	**422**	...
......				

FID	1	2	3	...
width	980	161	211	...
nchannel	3.1	1.0	1.4	...
sinuosity	1.1	1.1	1.1	...
PSI	19.6	15.2	4.8	...
PMI	13.0	31.6	0.5	...
PGS	31.5	45.5	77.1	...
PMD	8.7	3.9	13.8	...
PSD	26.9	3.6	3.7	...
...	...	...	...	...
PI-PD	3	39.3	12.2	...
**type**	**0**	**1**	**1**	...
......				

Fig. S2 | Flowchart of the key data processing steps. The detailed workflow is shown on the left, with illustrations of results on the right. (**a**) Retrieving statistical extent of rivers by using SWORD and a global lake inventory and identification of new reservoir-type river reaches (Type-R) with a global reservoir database. (**b**) Statistics of water extent changes and identification of morphological dynamics (Type-M) and hydrological signals (Type-H) at the level-9 basin scale by machine learning. (**c**) Analyzing patterns of water extent changes consisting of different types, focusing on the hotspots of Type-H rivers, and analysis of the driving forces. (**d**) Illustration of some results produced in the workflow (**a-c**), including river buffer zones, river change maps (percentage of significant increase on the Gan River, a tributary of Yangtze River as an example), as well as some thematic maps used for analysis.

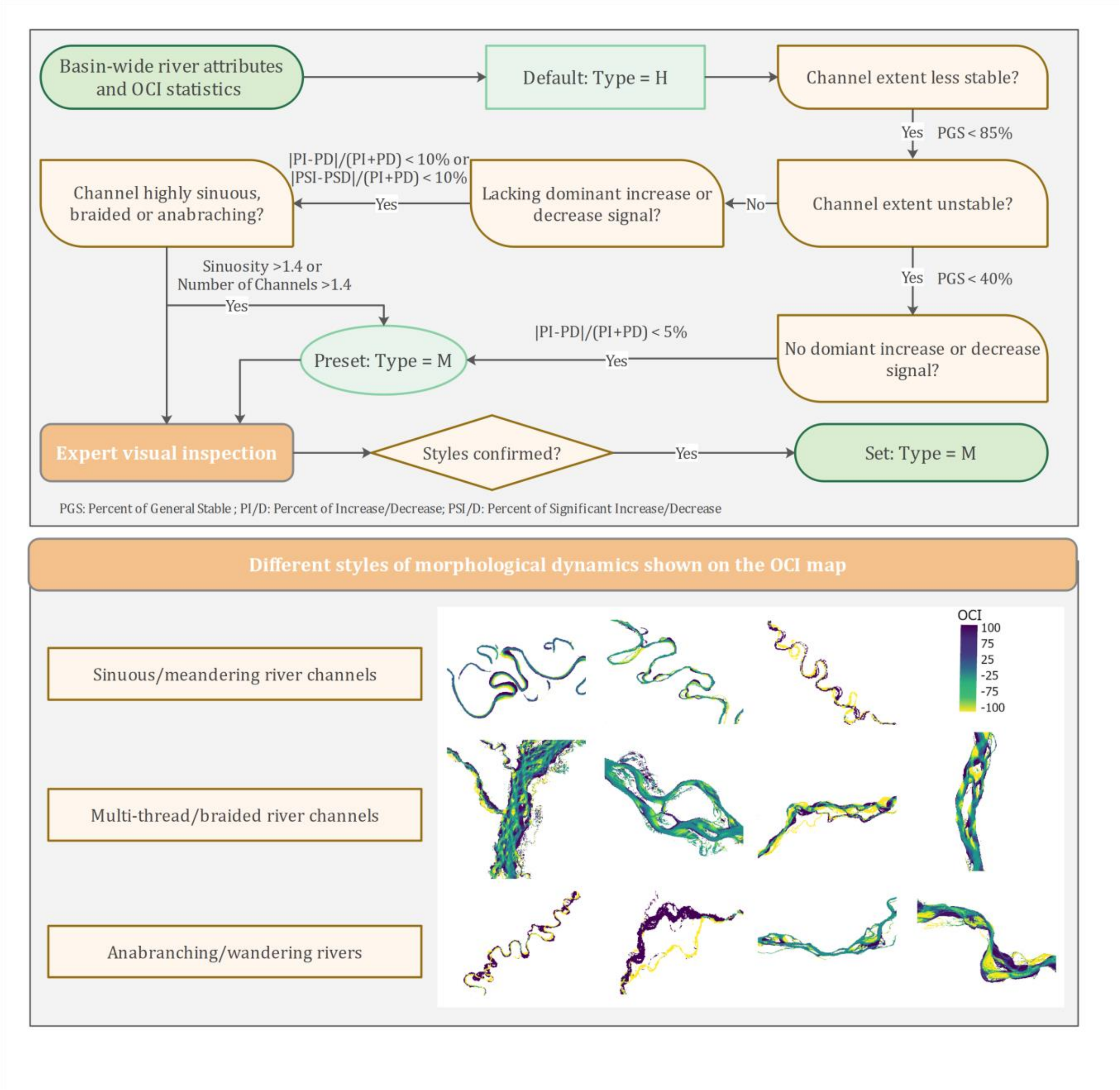

Fig. S3 | Expert-based protocol streamlining the procedure and standards for identifying Type-M (morphological dynamics) reaches from the machine learning training sample. The upper panel illustrates the decision tree guiding the separation of Type-M reaches from Type-H reaches in the training sample. The lower panel shows typical examples (in OCI) of different styles of morphological dynamics.

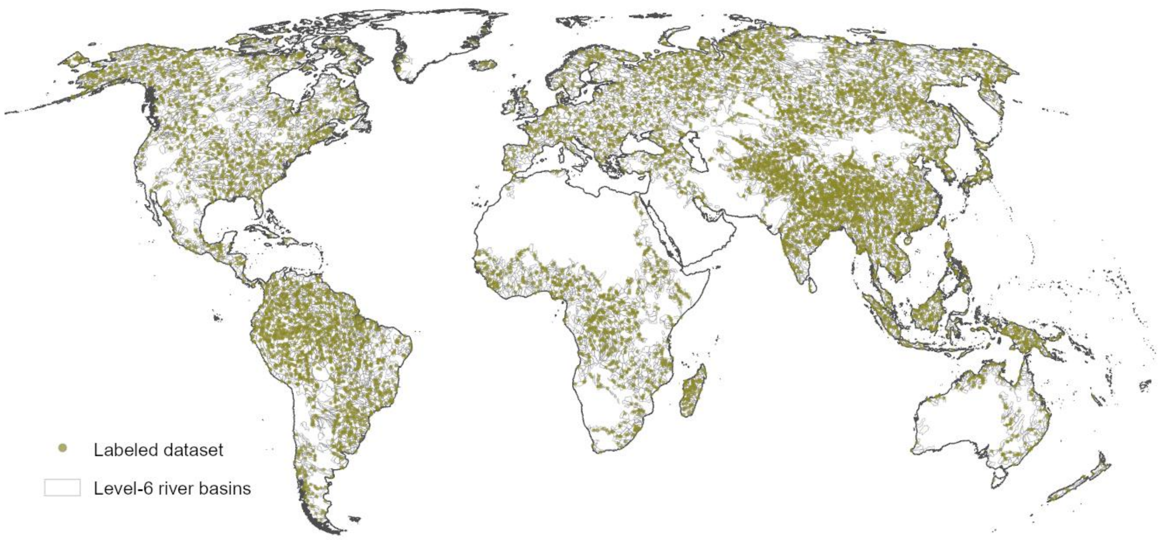

Fig. S4 | The distribution of sampled basins for manual identification of morphological dynamics (Type-M river reaches).

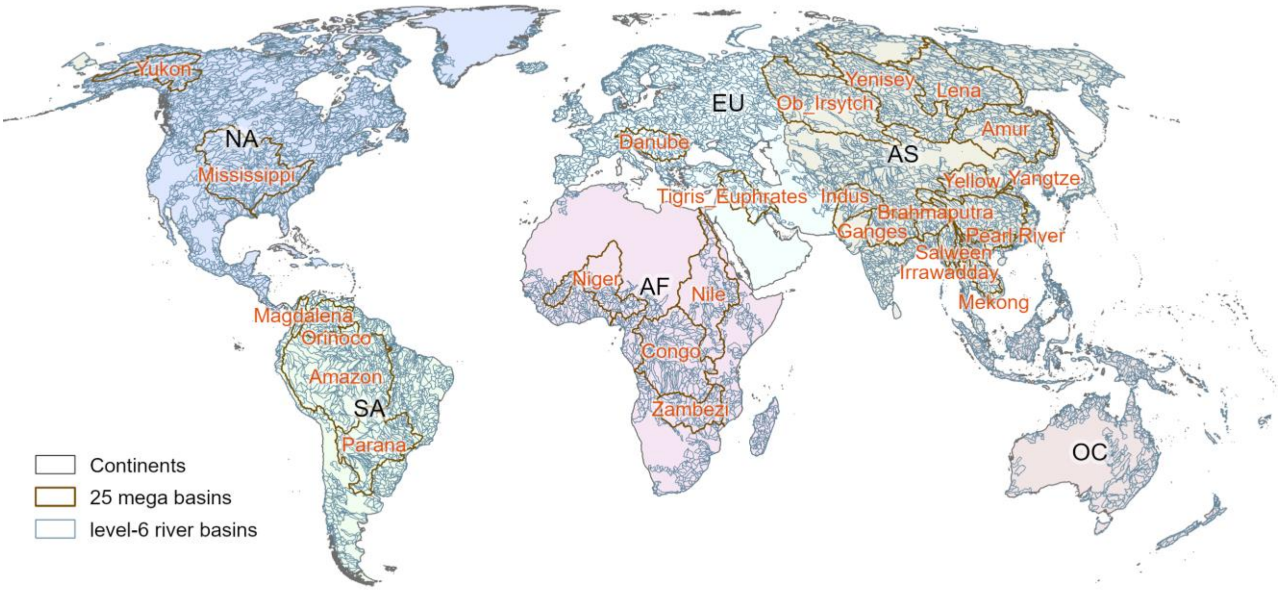

Fig. S5 | The division of continents, 25 mega basins, and level-6 river basins. HydroBASINS level-6 river basins are sourced from Lehner and Grill [3], and aggregated to 25 mega river basins according to Best [4]. Continent layer sourced from level-1 river basins of HydroBASINS dataset.

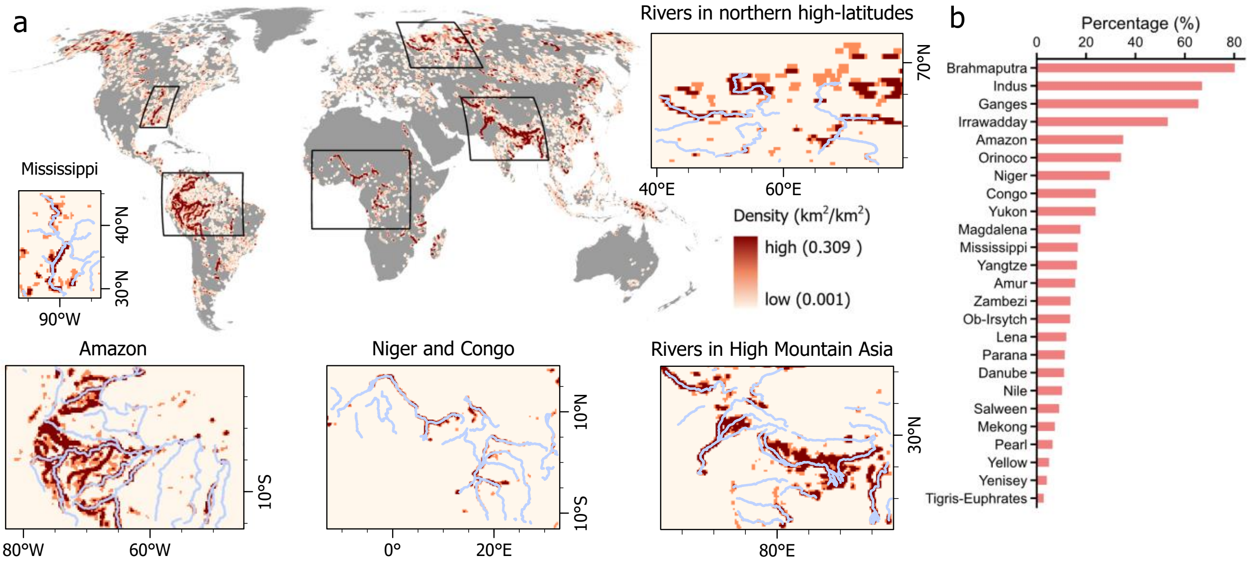

Fig. S6 | The distribution of Type-M river reaches (high morphological dynamics in the recent decades). (**a**)The kernel density map of Type-M basins (weighted by the river area) with insets showing zoom-in of some main regions. Blue lines represent major rivers. (**b**) The percentage of Type-M basins in each of the 25 mega basins (Fig. S5). In this statistic, the percentage represents the total area of Type-M river reaches divided by the total river area in the basin.

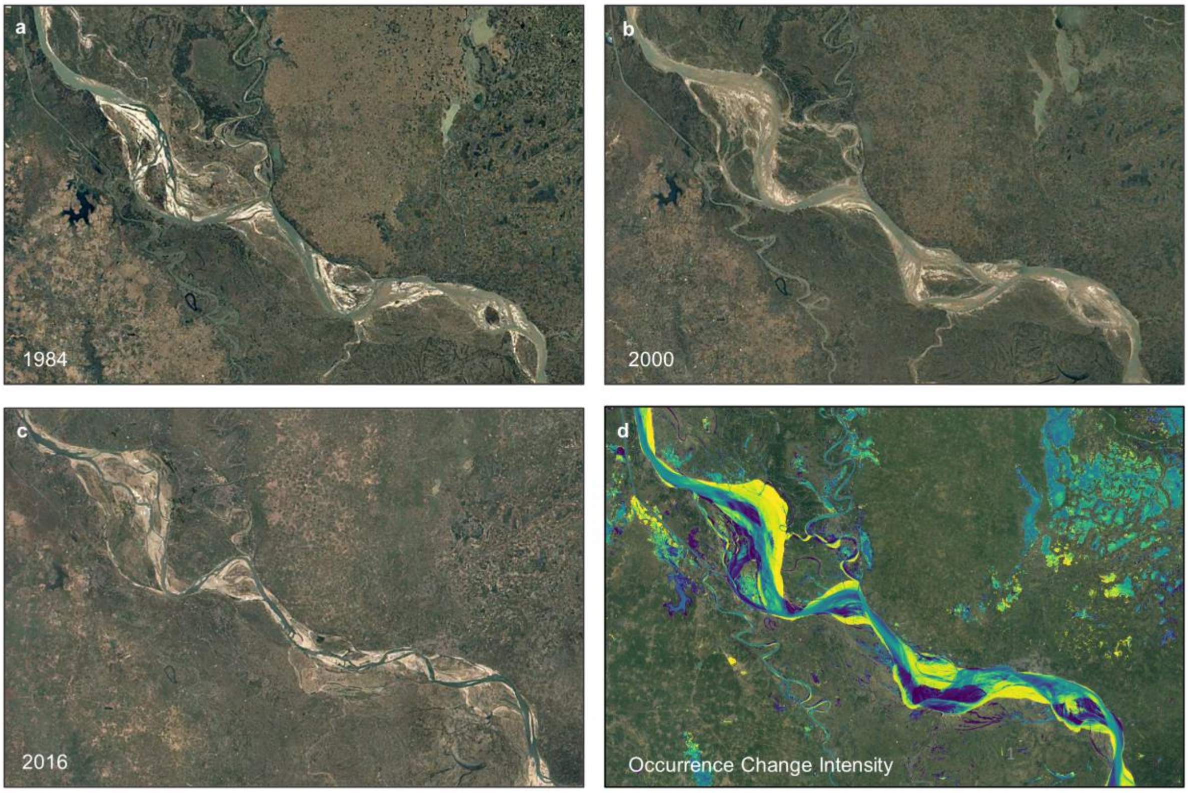

Fig. S7 | Fluvial morphology of a Brahmaputra River section. Fluvial morphology in 1984 (**a**), 2000 (**b**), and 2016 (**c**), and GSW OCI image showing river channel changes (**d**). The satellite images are obtained from the GSW website (https://global-surface-water.appspot.com/map).

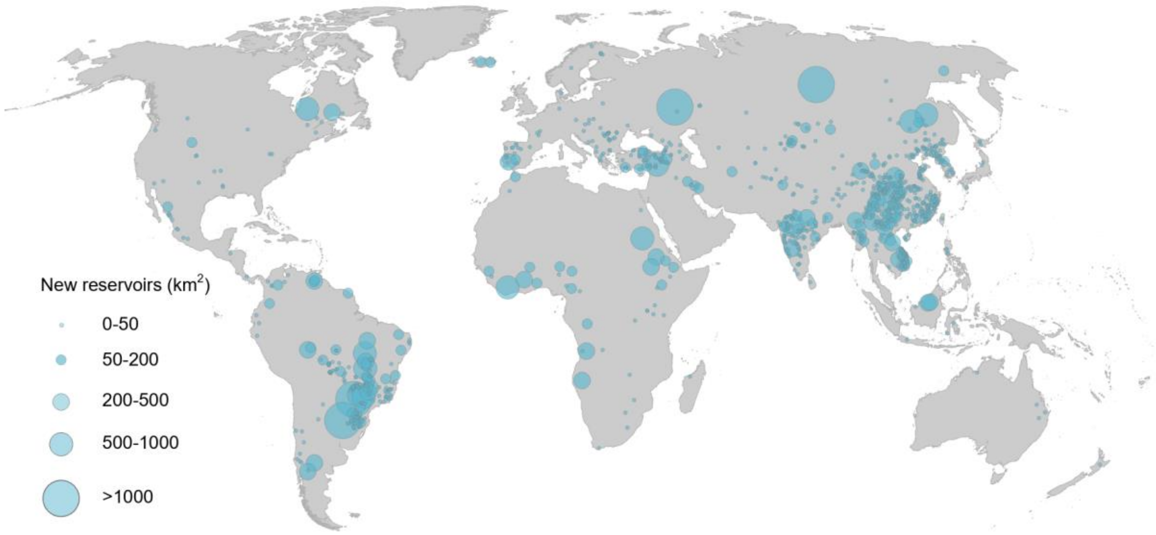

Fig. S8 | The distribution of new-dammed reservoirs on the SWORD river network.

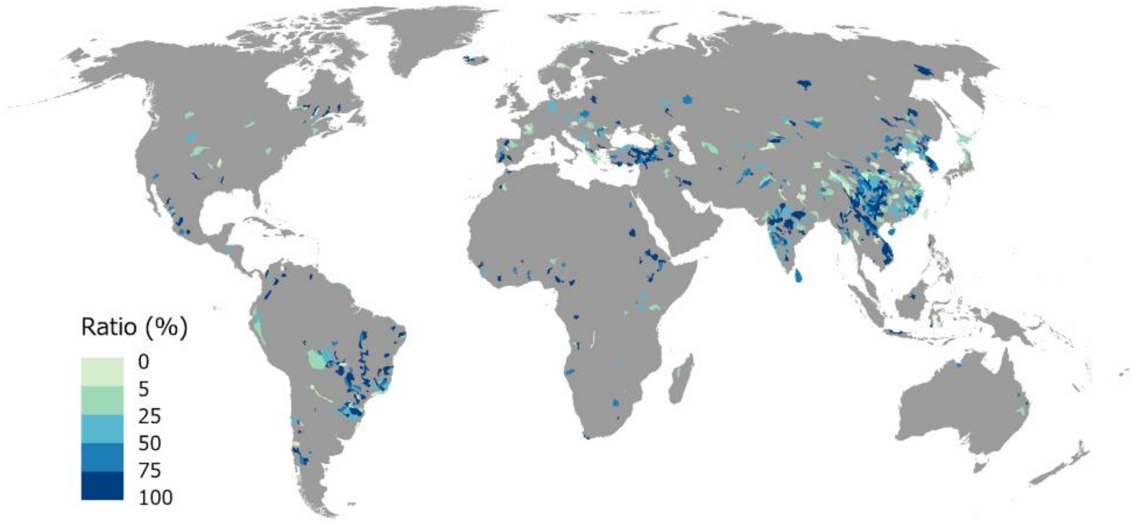

Fig. S9 | The contribution of dam-related widening to basin-wide total river widening. Here we define widening as frequency changes in the significant increase (SI) class. The ratio is the total area of SI class within the new reservoir-type rivers (Type-R) divided by all areas of SI class in the basin (Type-R and Type-H). The map reveals that new dams have been the major contributor to river widening in their basins.

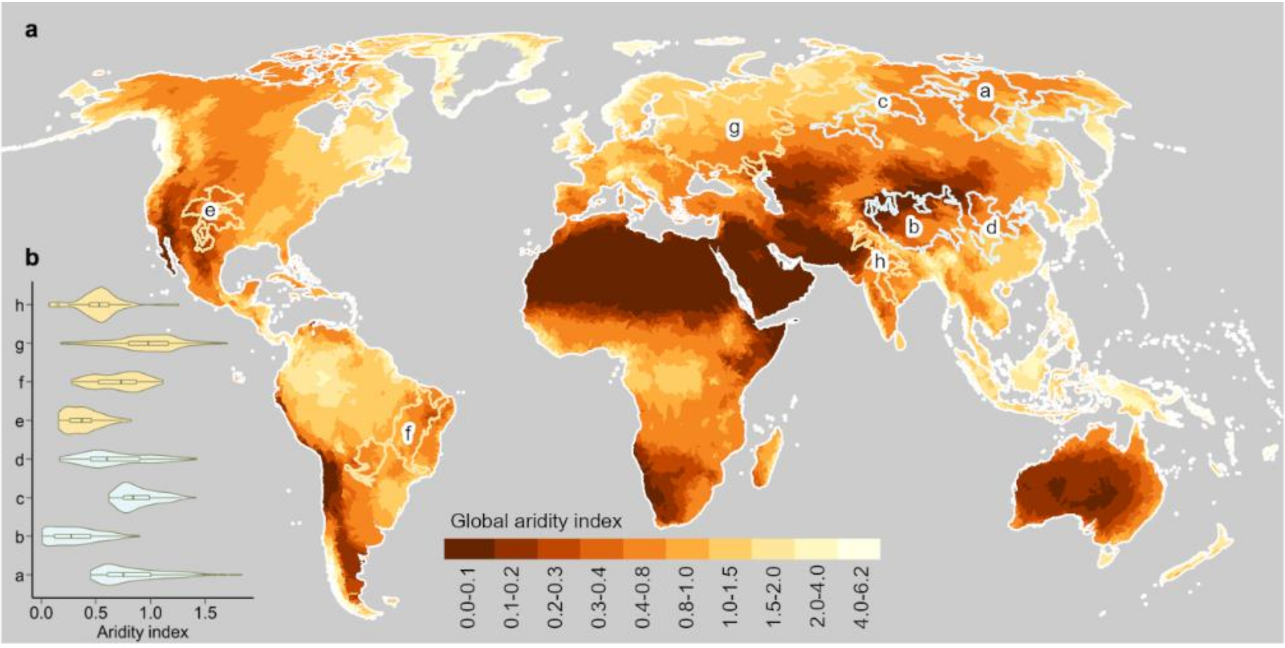

Fig. S10 | Global aridity index based on level-6 basins. (**a**) The global aridity index was computed as the ratio of the mean annual precipitation to the mean annual potential evapotranspiration. High aridity index indicates humid conditions. This dataset is sourced from Linke, Lehner [5] and Zomer, Trabucco [6]. (**b**) Statistics of basin-wide mean aridity in the eight hotspots of significant river changes. Light blue plots represent positive hotspots, and light-yellow plots represent negative hotspots.

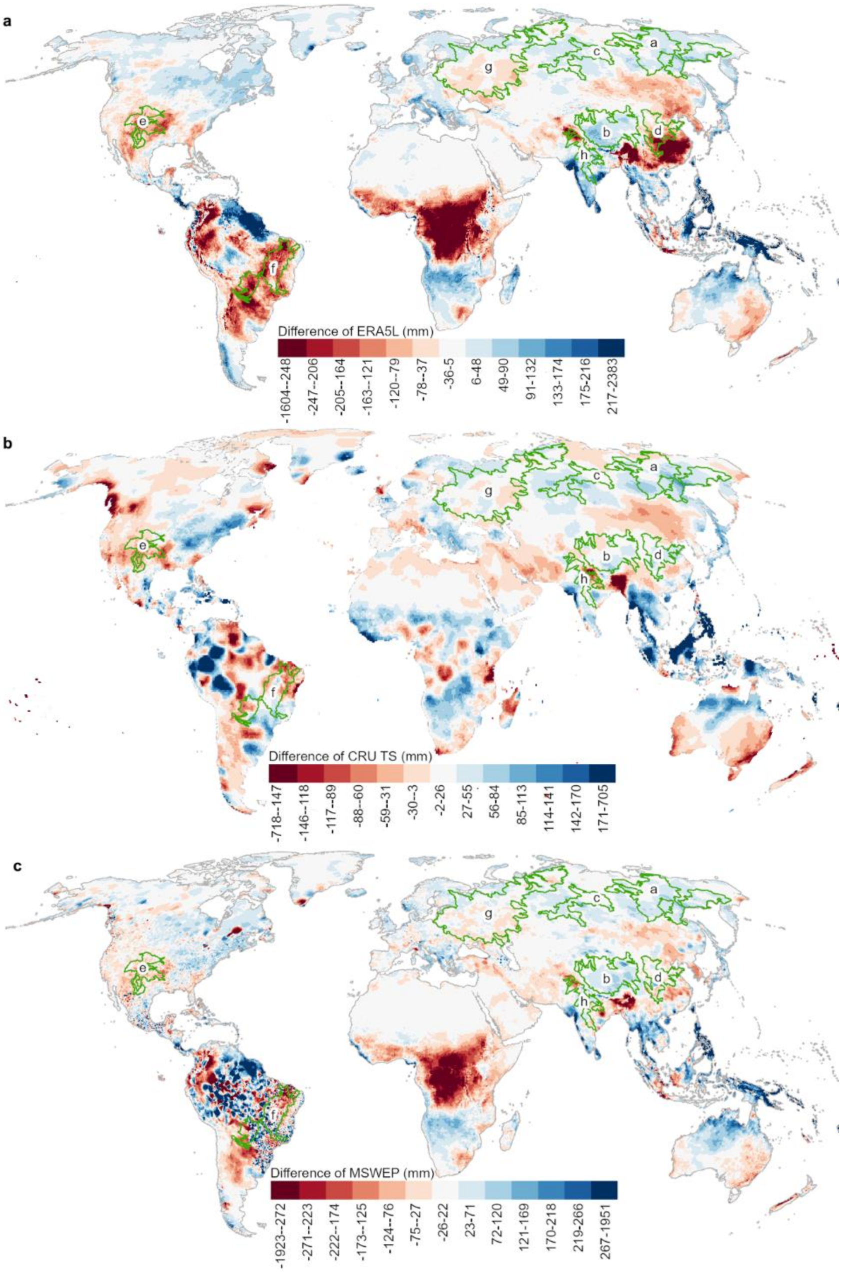

Fig. S11 | The difference between the mean annual precipitation between 1994-1999 and 2000-2018 from ERA5L, CRU TS, and MSWEP. The boundaries of four increased hotspots (a-d) and four decreased hotspots (e-h) are shown in green.

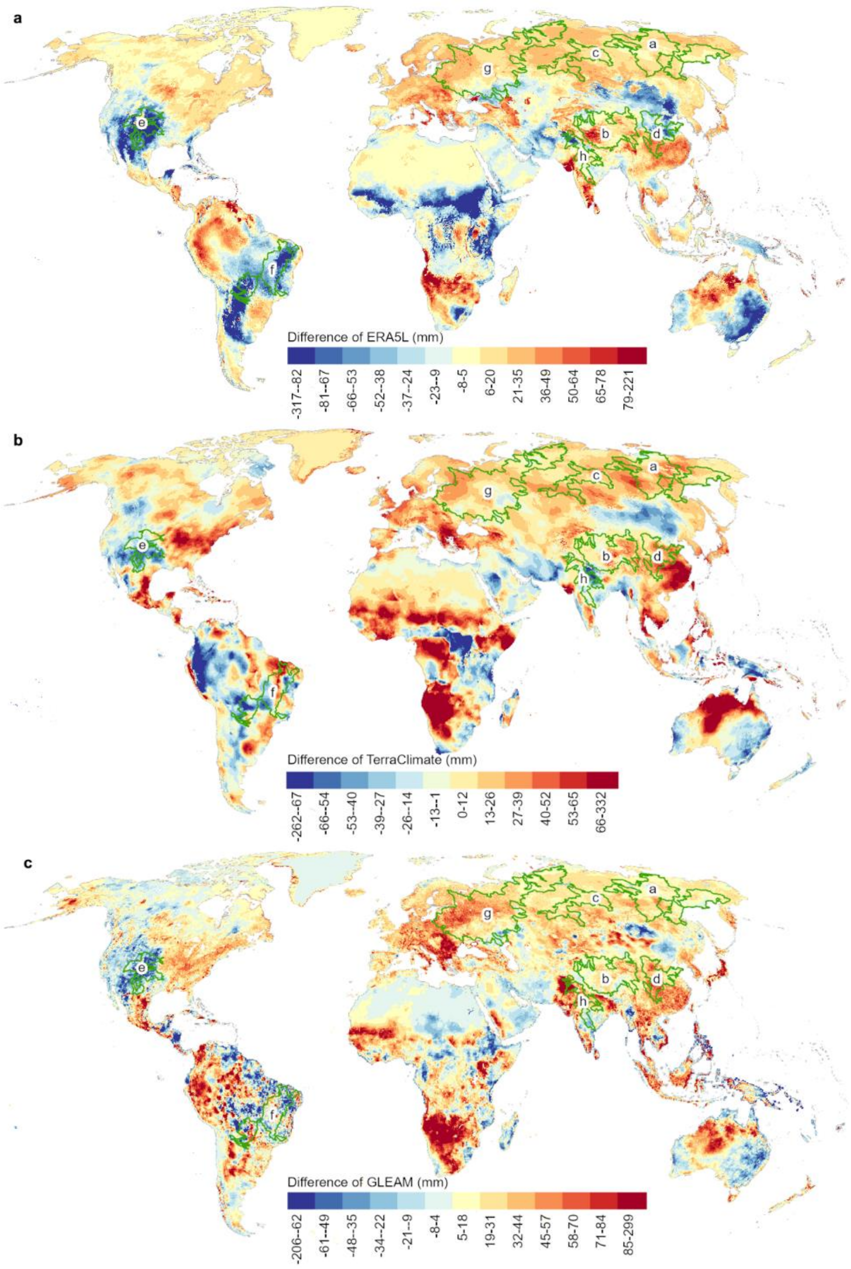

Fig. S12 | The difference between the mean annual evaporation between 1994-1999 and 2000-2018 from ERA5L, TerraClimate, and GLEAM. The boundaries of four increased hotspots (a-d) and four decreased hotspots (e-h) are shown in green.

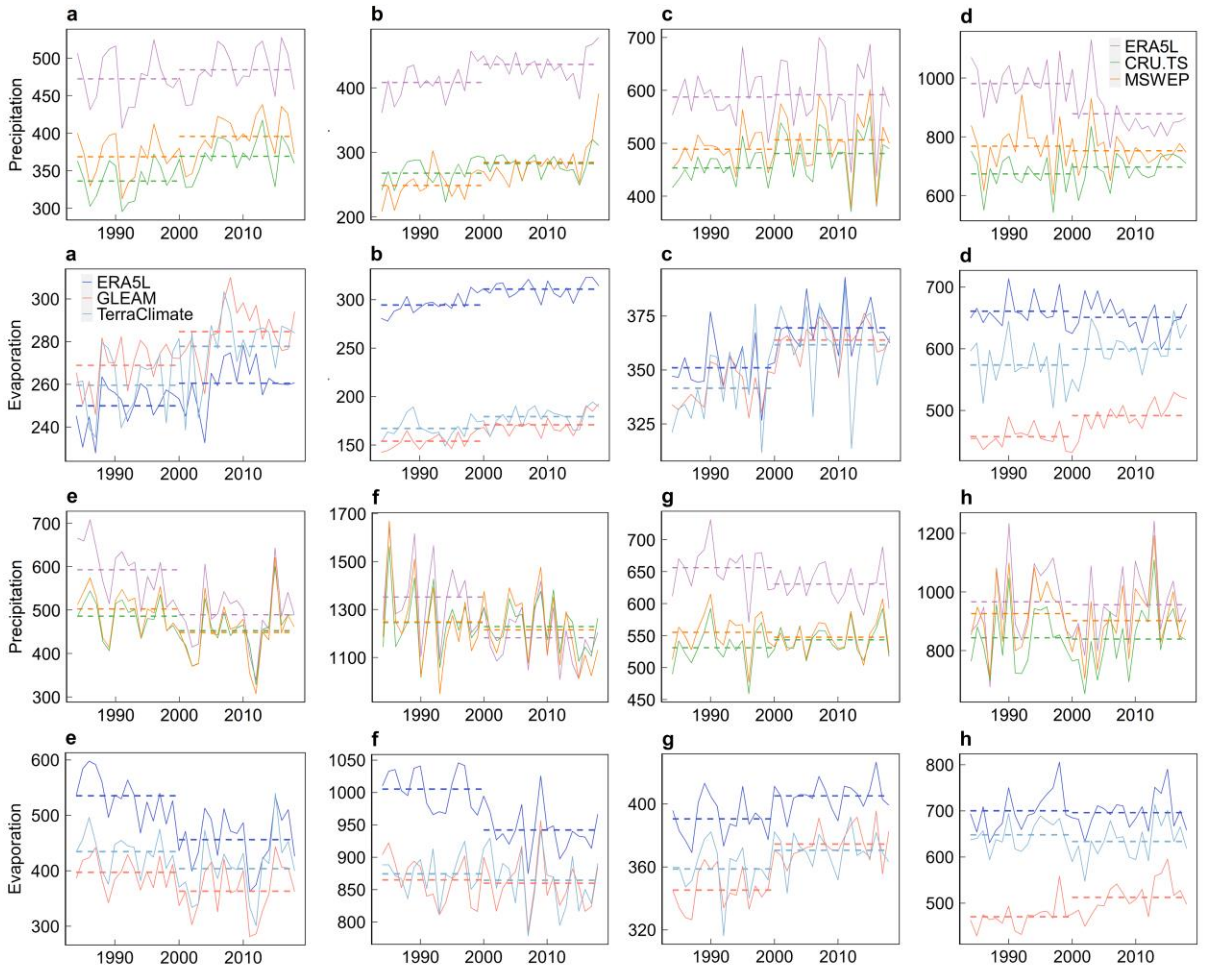

Fig. S13 | Eight hotspots' precipitation and evaporation trends during 1984-2018. The solid lines show the time series from 1984 to 2018, and the dashed lines represent averages for the two epochs. a-h labels the hotspots outlined in Fig. S10. Evaporation products include ERA5-Land, Climatic Research Unit gridded Time Series (CRU TS), and Multi-Source Weighted-Ensemble Precipitation (MSWEP) v2.8. Precipitation products include ERA5-Land, Global Land Evaporation Amsterdam Model (GLEAM) v3.6, and TerraClimate.

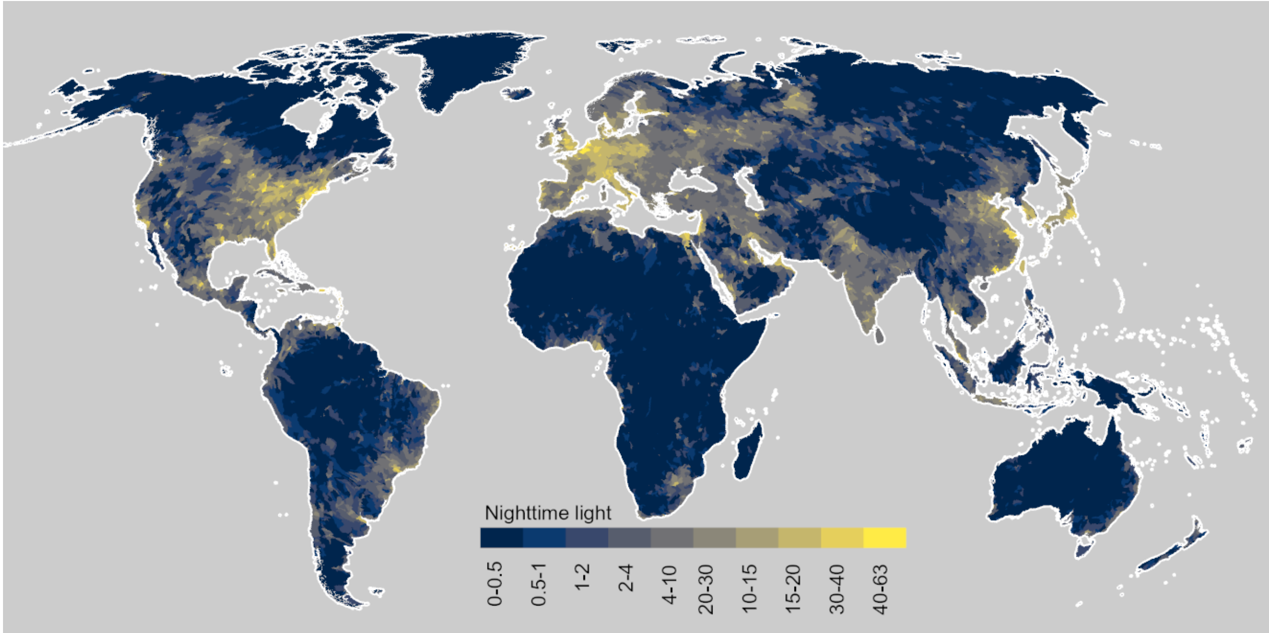

Fig. S14 | Global nighttime light for each level-6 basin. The nighttime light dataset represents light visibility generated by human activities. There is a strong correlation between nighttime light and PGS (Fig. 8), indicating that the stability of river channels is correlated with human activities (e.g., the construction of levees). This dataset is sourced from Doll [7] and re-formatted to level-6 basin scale in HydroATLAS database by Linke, Lehner [5].

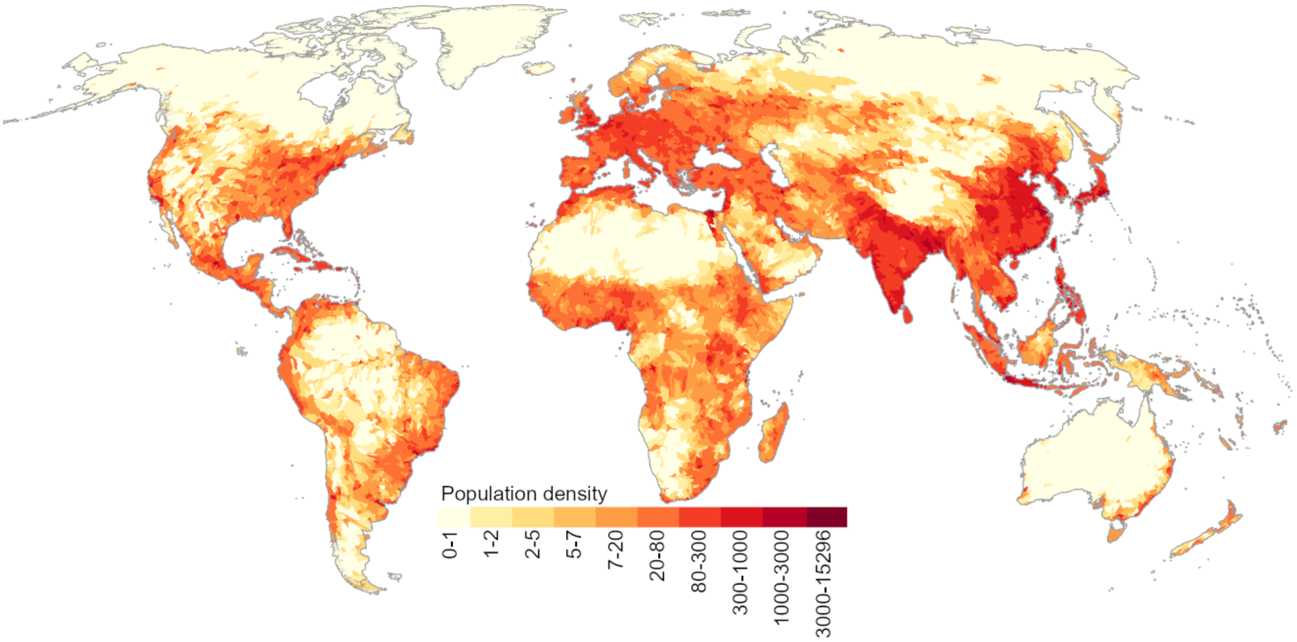

Fig S15. | The global population density map for each level-6 basin. The map is computed on the basis of population density map (number of persons per square kilometer, for the year 2000) provided by Center for International Earth Science Information Network - CIESIN - Columbia University [8] and aggregated to each level-6 basin. Basins with a mean population density less than 1 person/km^2 (in light yellow shade) were excluded in assessing the correlation between nighttime light and PGS.

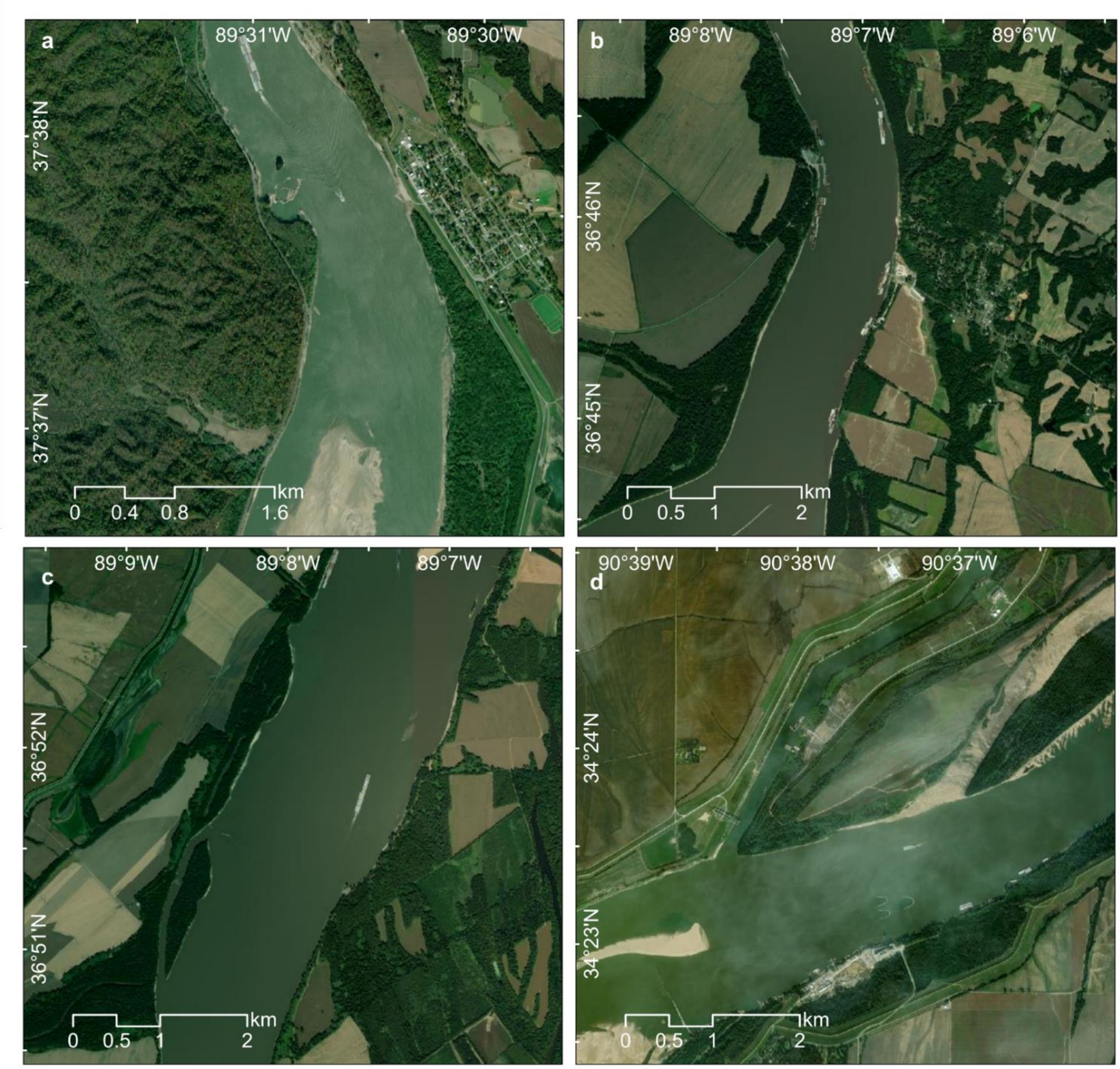

Fig. S16 | Levees along the Mississippi River. The river levees could be observed along most sections of the Mississippi River. The background image is the high-resolution imagery obtained from ESRI.

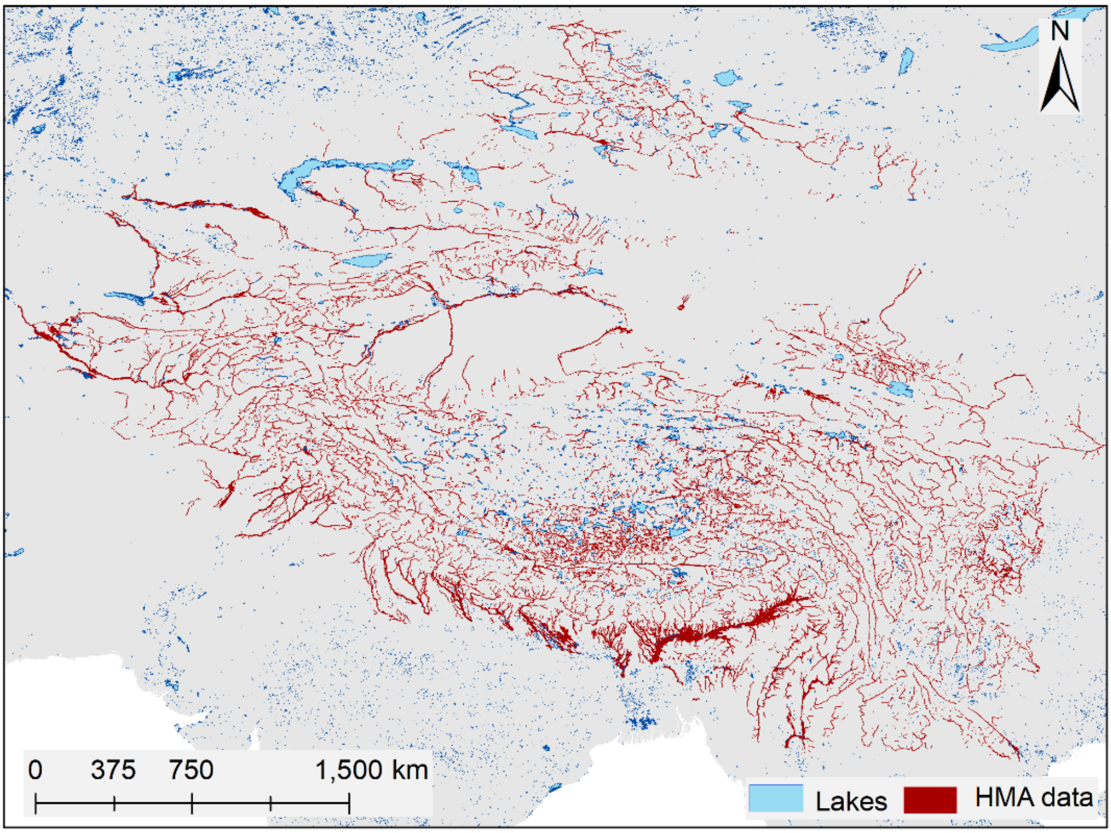

Fig. S17 | The river vector dataset for High Mountain Asia (HMA). The lake distribution map is from the HydroLAKES dataset.

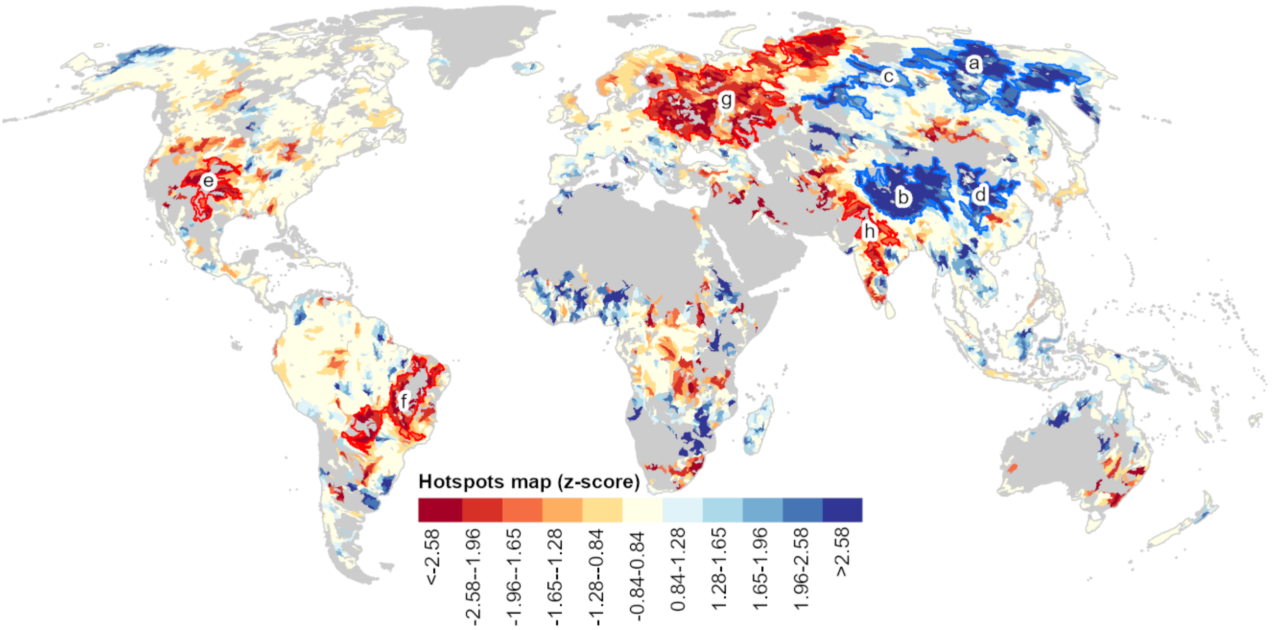

Fig. S18 | The spatial clusters of basins dominated by high river flow (increased hotspots) and those dominated by low river flow (decreased hotspots). The statistically significant clustering at the confidence level of 90% (z-score value of ± 1.65) is defined as the hotspots using the Hot Spot Analysis tool implemented in ArcGIS. The top four largest increased hotspots are located in Siberia (a), Tibetan Plateau (b), western Russia (c), and Yellow river basin (d), and the top four largest decreased hotspots are in central North America (e), eastern South America (f), western Europe (g), and India (h).

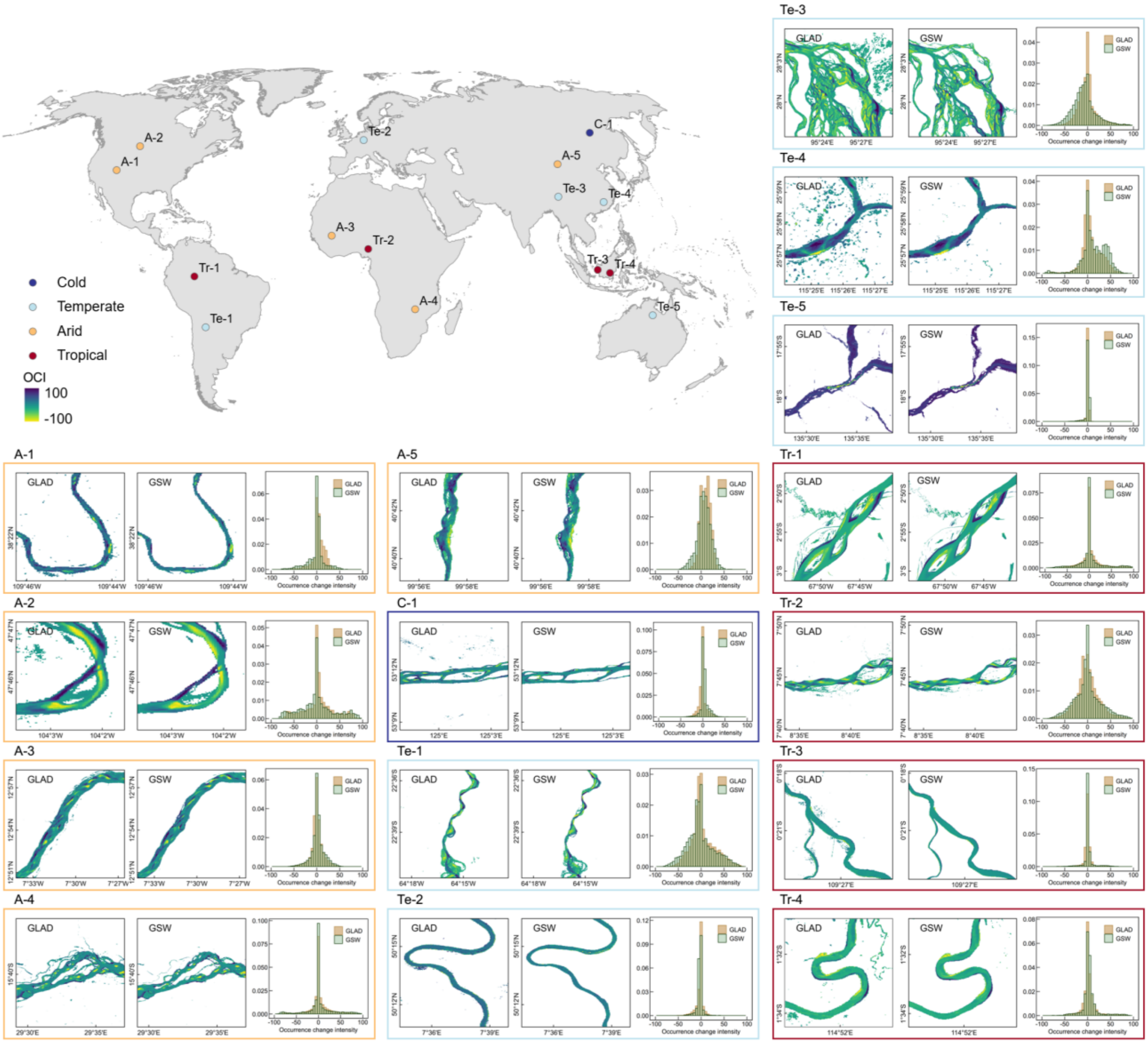

Fig. S19 | comparison of water frequency changes derived from the GSW OCI data with that from the Global Land Analysis and Discovery (GLAD) from 1999–2008 to 2009–2018, exemplified in different climate regions and different styles of river platforms and changes. For each case, the statistical distribution of frequency changes from each source is shown in the rightmost.

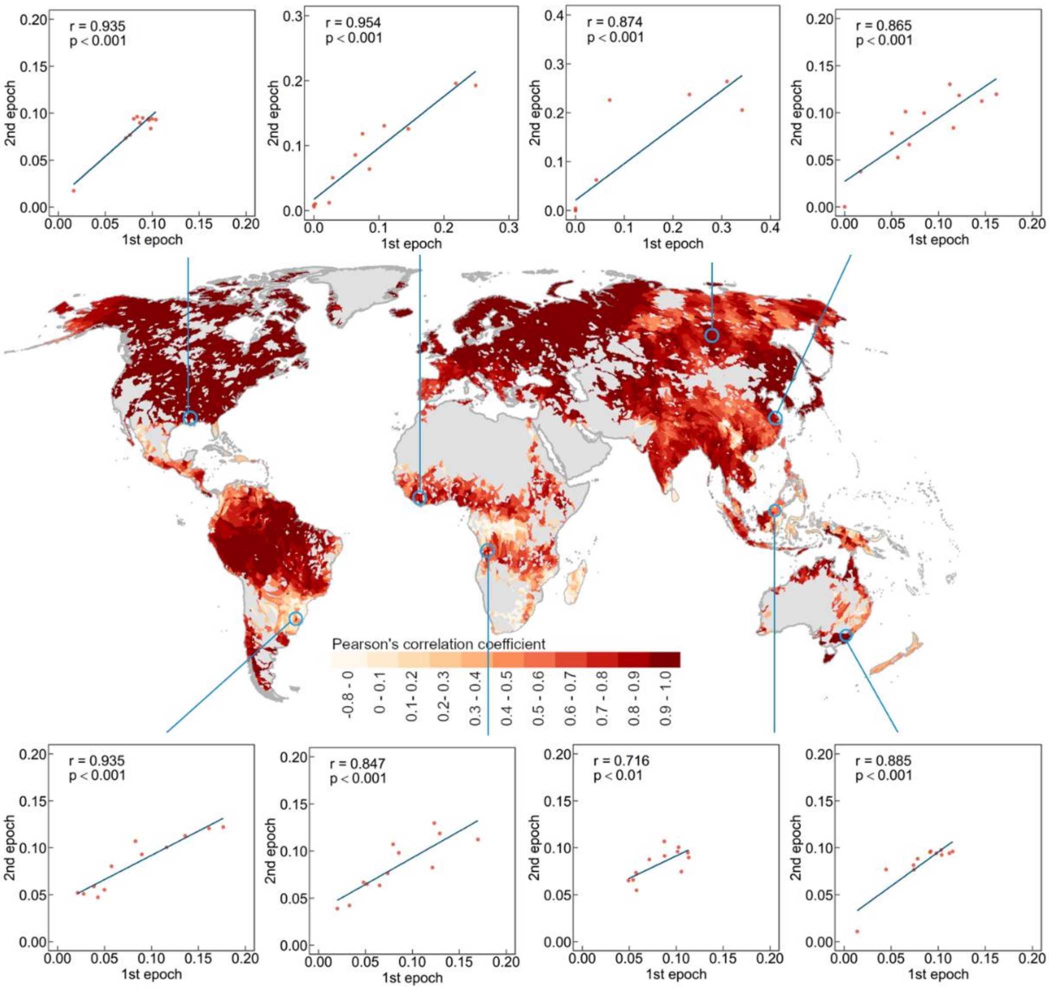

Fig. S20 | map of the Pearson's correlation coefficient between the total number of valid Landsat observations in each month during 1984-1999 and that during 2000-2018 in each studied level-6 river basins. High correlation coefficients indicate similar seasonal distributions on the coverage of Landsat imagery between the two epochs. The insets show eight basins illustrating the correlation in the seasonal distribution of observations. We counted the number of valid observations in each month during 1984-1999 and that of during 2000-2018 referring to the GSW monthly water history dataset on the Google Earth Engine platform. To compare the seasonal distribution of observations during the two epochs, we calculated the Pearson's correlation coefficient between the vector of monthly observations before and after 2000 in each level-6 river basin.

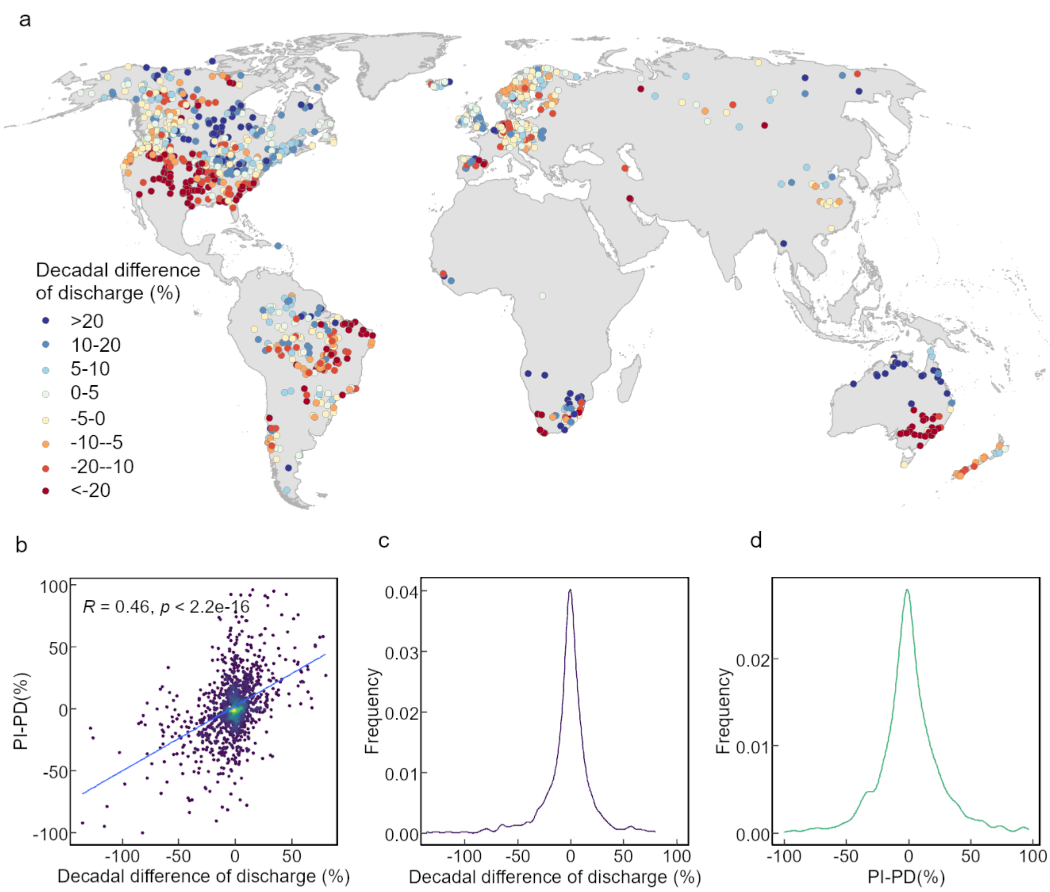

Fig. S21 | comparison between the statistics of the basin-wide river extent changes in this study and the discharge changes at gauging stations. (**a**)The distribution of gauging stations and the relative decadal difference of annual discharge; (**b**) the correlation between the difference of PI (percentage of increase) and PD (percentage of decrease) in Type-H basins (PI−PD) and relative decadal difference of discharge at the gauging station within the basin; (**c**) the frequency histogram of decadal difference of discharge, and (**d**) the frequency histogram of PI−PD. The long-term discharge data (1990−2018) were collected from Global Runoff Data Centre (GRDC) (https://www.bafg.de/GRDC/EN/Home/homepage_node.html) and related literature[9, 10, 11]. We only considered gauging stations located within the Type-H river basins. The relative decadal difference of discharge refers to the difference between the mean annual runoff during 1984 (or starting years no later than 1990) and 1999 and that of during 2000 and 2018 (2015−2018, due to different temporal spans of gauging records) divided by the mean annual runoff over the entire period.

Table S1 | The total river area and the percentage of significantly decreased (PSD), moderately decreased (PMD), general stable (PGS), moderately increased (PMI), and significantly increased (PSI) frequency of Type-H river reaches in the 25 representative basins in Africa, Eurasia, North America, and South America. The bold font highlights higher values in the lists of PI (PSI+PMI) and PD (PSD+PMD).

Continent	River Name	PSD (%)	PMD (%)	PGS (%)	PMI (%)	PSI (%)	Area (km^2)	PD (%)	PI (%)
Africa	Congo	6.08	6.22	78.69	5.53	3.49	11744.7543	12.29	9.02
	Nile	5.27	4.41	65.11	21.57	3.64	9581.0715	9.68	25.21
	Niger	4.82	6.04	57.12	18.33	13.68	7686.6291	10.87	32.01
	Zambezi	1.24	1.33	76.37	12.85	8.22	9886.2813	2.57	21.06
Eurasia	Ob-Irsytch	3.70	9.64	67.21	9.01	10.44	23465.1870	13.34	19.45
	Yenisey	2.28	3.91	84.71	4.44	4.65	19669.0185	6.20	9.10
	Yangtze	9.60	9.04	56.15	12.26	12.95	14347.8846	18.64	25.21
	Amur	3.33	7.03	62.02	19.04	8.57	17444.5047	10.37	27.61
	Ganges	10.81	10.53	52.66	11.27	14.73	9271.1430	21.34	26.00
	Lena	1.59	4.05	77.79	4.70	11.87	13581.8712	5.64	16.56
	Brahmaputra	11.24	9.90	31.36	20.36	27.13	3055.0743	21.14	47.50
	Indus	14.51	14.63	50.50	9.20	11.15	3937.1616	29.14	20.35
	Mekong	2.62	5.41	59.96	18.22	13.78	7232.2695	8.04	32.00
	Irrawaddy	9.09	17.24	39.78	15.21	18.68	3108.0996	26.32	33.89
	Tigris/Euphrates	10.25	8.83	71.46	6.32	3.14	4657.0293	19.08	9.47
	Yellow River	7.08	4.40	22.97	22.27	43.28	3423.0690	11.48	65.55
	Danube	10.81	10.53	52.66	11.27	14.73	9271.1430	21.34	26.00
	Pearl River	4.96	6.36	65.88	9.60	13.20	2109.6009	11.32	22.80
	Salween	3.76	4.85	63.12	13.36	14.89	1011.8025	8.62	28.26
North America	Mississippi	4.05	7.57	77.27	7.17	3.95	22305.1140	11.62	11.11
	Yukon	3.13	4.48	75.76	6.23	10.40	5589.9387	7.61	16.63
South America	Amazon	4.30	4.64	71.69	9.37	9.99	47856.4794	8.95	19.36
	Parana River	8.36	11.62	66.02	4.86	9.13	23795.6796	19.98	13.99
	Magdalena	11.36	7.45	31.13	19.21	30.85	1909.8090	18.82	50.05
	Orinoco	5.82	7.13	63.22	10.72	13.12	7456.8627	12.95	23.84

Table S2 | Eight large lake-type reservoirs included in the HydroLAKES dataset. The following lake-type reservoirs are included in our statistics with the following considerations: (1) the lake type is reservoir, (2) lake area ≥100 km^2, and (3) shoreline development (i.e., the ratio between shoreline length and the circumference of a circle with the same area) ≤3.

Lake name	Country	Continent	Lake area (km^2)	Shoreline development
**Victoria**	Australia	Oceania	107.51	1.17
**Red Lake Reservoir**	United States of America	North America	1141.61	1.73
**Mari Menuco**	Argentina	South America	163.90	1.73
**Kayrakkumskoye**	Turkmenistan	Asia	153.65	1.91
**Canyon**	United States of America	North America	102.87	2.01
**Lake Maga**	Cameroon	Africa	115.94	2.16
**Utah Lake**	United States of America	North America	362.19	2.24
**Los Barreales**	Argentina	South America	395.81	2.46

Table S3 | Precision, recall, overall accuracy, the kappa coefficient, and the F1-score of the confusion matrix with the XG-Boost, Random Forest (RF), and Feedforward Neural Network (FNN) model.

	Accuracy(%)	Kappa(%)	Precision(%)	Recall(%)	F1-score(%)
**XG-Boost**	92.23	76.83	94.08	96.07	95.06
**RF**	91.93	75.28	94.65	95.19	94.92
**FNN**	89.99	68.56	93.49	94.01	93.75

Table S4 | The evaluation metrics for different continents based on the XG-Boost model.

	Accuracy(%)	Kappa(%)	Precision(%)	Recall(%)	F1-score(%)
**Africa**	96.50	89.05	92.86	89.66	91.23
**Asia**	94.34	81.47	82.29	87.78	84.95
**Europe**	93.24	79.69	96.30	74.29	83.87
**North America**	94.12	82.57	89.13	83.67	86.32
**Oceanic**	96.15	87.30	86.67	92.86	89.66
**South America**	94.51	85.83	90.91	88.24	89.55
**Global**	94.55	83.59	88.13	85.96	87.03

References

1. Pickens AH, *et al.* Mapping and sampling to characterize global inland water dynamics from 1999 to 2018 with full Landsat time-series. *Remote Sensing of Environment* **243**, 111792 (2020).

2. Pekel J-F, Cottam A, Gorelick N, Belward AS. High-resolution mapping of global surface water and its long-term changes. *Nature* **540**, 418-422 (2016).

3. Lehner B, Grill G. Global river hydrography and network routing: baseline data and new approaches to study the world's large river systems. *Hydrological Processes* **27**, 2171-2186 (2013).

4. Best J. Anthropogenic stresses on the world’s big rivers. *Nature Geoscience* **12**, 7-21 (2019).

5. Linke S, *et al.* Global hydro-environmental sub-basin and river reach characteristics at high spatial resolution. *Scientific Data* **6**, (2019).

6. Zomer RJ, Trabucco A, Bossio DA, Verchot LV. Climate change mitigation: A spatial analysis of global land suitability for clean development mechanism afforestation and reforestation. *Agriculture, Ecosystems & Environment* **126**, 67-80 (2008).

7. Doll CNH. CIESIN Thematic Guide to Night-time Light Remote Sensing and its Applications. (2008).

8. Center for International Earth Science Information Network - CIESIN - Columbia University. Gridded Population of the World, Version 4 (GPWv4): Population Density, Revision 11.). NASA Socioeconomic Data and Applications Center (SEDAC) (2018).

9. Liu F, Xie R, Luo X, Yang L, Cai H, Yang Q. Stepwise adjustment of deltaic channels in response to human interventions and its hydrological implications for sustainable water managements in the Pearl River Delta, China. *Journal of Hydrology* **573**, 194-206 (2019).

10. Wu Z, Milliman JD, Zhao D, Cao Z, Zhou J, Zhou C. Geomorphologic changes in the lower Pearl River Delta, 1850–2015, largely due to human activity. *Geomorphology* **314**, 42-54 (2018).

11. Gao Y, Chen L, Zhang W, Li X, Xu Q. Spatiotemporal variations in characteristic discharge in the Yangtze River downstream of the Three Gorges Dam. *Science of the Total Environment* **785**, 147343 (2021).
